# Physical working conditions and subsequent sickness absence: a record linkage follow-up study among 19–39-year-old municipal employees

**DOI:** 10.1007/s00420-021-01791-y

**Published:** 2021-10-23

**Authors:** M. Mänty, A. Kouvonen, H. Nordquist, J. Harkko, O. Pietiläinen, J. I. Halonen, O. Rahkonen, T. Lallukka

**Affiliations:** 1 Department of Public Health, University of Helsinki, Helsinki, Finland; 2 Unit of Strategy and Research, City of Vantaa, Vantaa, Finland; 3 Faculty of Social Sciences, University of Helsinki, Helsinki, Finland; 4 Centre for Public Health, Queen’s University Belfast, Belfast, UK; 5 Department of Health Care and Emergency Care, South-Eastern Finland University of Applied Sciences, Kotka, Finland; 6 Department of Health Security, Finnish Institute for Health and Welfare, Helsinki, Finland

**Keywords:** Cohort study, Sick leave, Young employees, Public sector, Occupational exposures

## Abstract

**Purpose:**

Physical work exposures are associated with sickness absence among older employees. We aimed to examine if they similarly contribute to all-cause sickness absence during early and mid-careers.

**Methods:**

We used questionnaire data on physical work exposures linked to register data on sickness absence from 3542 municipal employees aged 19–39 years. Follow-up for the number of sickness absence days was 12 months. Exposures to physical workload, occupational environmental hazards, and sedentary work were divided into quartiles. In addition, duration of daily exposure to heavy work was included. Negative binomial regression models were used.

**Results:**

Higher exposure to physical workload or hazardous exposures was associated with a higher number of sickness absence days. The age and gender adjusted rate ratios for sickness absence days among the participants whose exposure to physical workload was in the highest exposure quartile were 2.1 (95% CI 1.8‒2.5) compared with those whose exposure was in the lowest quartile. In addition, rate ratios for sickness absence days among participants who reported that they do heavy physical work 1.1‒2.0 h, 2.1‒4.0 h or over 4 h daily were 1.6 (1.3‒1.9), 1.5 (1.3‒1.8) and 1.7 (1.5‒2.1), respectively, compared with those who reported not doing physical work. Further adjustment for lifestyle factors or health characteristics attenuated the associations only slightly.

**Conclusion:**

Exposure to physically demanding work is associated with a higher number of sickness absence days among municipal employees below 40 years of age. Physical working conditions should be considered when aiming to support later work ability.

**Supplementary Information:**

The online version contains supplementary material available at 10.1007/s00420-021-01791-y.

## Introduction

Work disability, namely, sickness absence and disability pension, is a major social and economic problem throughout the Organisation for Economic Co-operation and Development (OECD) countries (OECD 2010). Sickness absence denotes temporal absence from work due to transient inability to perform one´s tasks at work as a consequence of disease or injury. Moreover, sickness absence reflects ill health and poor health-related functioning (Marmot et al. 1995; Kivimäki et al. 2003; Laaksonen et al. 2011), and predicts future permanent work disability (Kivimäki et al. 2004; Lund et al. 2008). Recent studies using register data have shown that sickness absence is highly prevalent already during early work careers (Sumanen et al. 2015a, 2017a). However, studies based solely on register data are unable to examine potential modifiable determinants of sickness absence, such as working conditions. To be able to target interventions to prevent sickness absence and subsequent more severe work disability, it is crucial to understand the factors contributing to work disability already at early and mid-career stages.

Previous studies have shown that the incidence of sickness absence and predictors of work disability vary by age (Krane et al. 2014; Sumanen et al. 2015b; Ervasti et al. 2017). For example, earlier results have indicated that the association between low job control and all-cause work disability becomes stronger with increasing age, and that the association between chronic diseases and work disability is stronger among younger than among older employees (Ervasti et al. 2017). In addition, even if older employees have more frequent long periods of sickness absence than their younger counterparts, younger employees tend to have more frequent short sickness absence periods (Taimela et al. 2007; Sumanen et al. 2015b). Frequent short periods of sickness absence have been shown to predict subsequent longer absence (Hultin et al. 2012; Laaksonen et al. 2013; Sumanen et al. 2017b) and thus, sickness absence at an early career stage may be an alarming sign of declining health and work ability.

Evidence has accumulated showing that modifiable work-related factors, such as psychosocial and physical working conditions, play an important role in the development of work disability (Lund et al. 2006; Head et al. 2006; Christensen et al. 2008; Laaksonen et al. 2010b; Halonen et al. 2021). For example, earlier studies among midlife and older employees have shown that many physical characteristics of work are associated with poorer health (Mänty et al. 2015; Kouvonen et al. 2016, 2017; Halonen et al. 2021) and increased risk of work disability, namely, sickness absence (Lund et al. 2006; Laaksonen et al. 2010b; Andersen et al. 2016; Halonen et al. 2021). Moreover, long-term and cumulative exposure to heavy physical work has been associated with disability retirement (Lahelma et al. 2012; Ervasti et al. 2017, 2019) and even premature mortality (Ervasti et al. 2019). However, we are not aware of studies that would have examined whether physical working conditions are associated with sickness absence specifically among younger employees.

Thus, this study aims to examine the association between physical working conditions and subsequent all-cause sickness absence among 19–39-year-old municipal employees.

## Methods

### Participants

This study is part of the Young Helsinki Health Study (Lallukka et al. 2020b) that examines the health and well-being among the young employees of the City of Helsinki, Finland. The Young Helsinki Health Study is an extension to the Helsinki Health Study, a cohort study following midlife and older employees of the City of Helsinki (Lahelma et al. 2013), and it covers a wide range of different occupations from manual workers to routine non-manual staff, professionals and managers from various sectors, such as health and social care, education, and culture. The data collection was conducted mainly via online and mailed questionnaires in autumn 2017. In addition, telephone interviews were conducted to target those who did not respond online or via mail. The original target population included 11,459 young employees (18‒39 years of age) of the City of Helsinki, who were born in 1978 or later, who had a job contract of at least 50% of regular work hours per week and whose employment contract had lasted at least 4 months before the data collection began. These criteria were applied to exclude those still on probation and those working only few hours a week (Lallukka et al. 2020b). Overall, the response rate was 51.5% (*n* = 5 898). Although the participants have been shown to represent the target population fairly well, those with a higher socioeconomic position and less long-term sickness absence were somewhat more likely to respond to the survey (Lallukka et al. 2020b). The survey data were prospectively linked to employer´s personnel register data on sickness absence Consent to the linkage was provided by 82% (*n* = 4 864) of the participants.

As the telephone interview included only a small set of variables, we included only the participants who responded to the online or mailed surveys (*n* = 5 111). In addition, only those who gave permission to link their survey responses with the register data (*n* = 4 213), who reported being currently full- or part-time employed (*n* = 3 769) and who had full information on all the variables used (*n* = 3 542) were included in the analyses. We did not make any exclusions based on previous episodes of sickness absence. Participants in the final analytical sample (*n* = 3 542) are likely to be comparable with all the respondents who gave their permission to register linkages (*n* = 4 864) as they had the same amount of sickness absence days during the 12-month post-survey follow-up: 20.9 days (95% CI 19.9–22.0) among the included vs 21.2 days (95% CI 20.2–22.4) among all who gave permission.

The study was approved by the City of Helsinki and Faculty of Medicine, University of Helsinki ethics committee, Finland.

### Physical work exposures

The survey included an 18-item inventory regarding different physical work exposures (Piirainen et al. 2003). Participants were asked whether they were exposed to each condition and to what extent it bothered them with the response alternatives: “does not exist”, “exists but does not bother”, “exists and somewhat bothers” and “exists and bothers a lot”. Following the procedures used also in our previous studies (Laaksonen et al. 2010b; Mänty et al. 2016; Halonen et al. 2021), three broad factors were obtained by factor analysis: physical workload, sedentary work, and occupational environmental hazards. Six items loaded on the *physical workload* factor: uncomfortable postures, repetitive trunk rotation, repetitive movements, heavy physical work, standing and walking (Cronbach *α* = 0.83). Three items loaded on the *sedentary work*: working with a computer display terminal, using a computer mouse and doing sedentary work (Cronbach *α* = 0.82). The third factor, that is, occupational *environmental hazards*, comprised exposures to dirt and dust, dampness, noise, solvents or other irritating substances, and problems with lighting or temperature (Cronbach *α* = 0.76). In each of the factors, a high score indicates high exposure and participants were stratified into quartiles based on their factor scores: 1 (lowest quartile = lowest exposure) – 4 (highest quartile = highest exposure).

In addition to various physical work exposures, *duration of daily exposure to heavy work* was assessed. Participants were asked to evaluate how many hours and minutes of physically demanding work (e.g., tasks including heavy lifting or climbing stairs) they do on average per day. Participants were stratified into five categories based on the duration of exposure: (1) 0 h, (2) 0.1‒1.0 h, (3) 1.1‒2.0 h, (4) 2.1‒4.0 h and (5) over 4 h per day.

### Sickness absence outcome

The questionnaire-based baseline data was prospectively linked with employer´s personnel register data on sickness absence. The register covers sickness absence spells of all employees to an accuracy level of 1 day. The number of sickness absence days was followed from the return of the questionnaire for 12 months or until the end of the employment contract, whichever came first. The number of sickness absence days was used as a count data outcome.

### Covariates

The baseline questionnaire collected information on sociodemographic (age and gender) and health characteristics (chronic conditions, pain, obesity and sleep problems) as well as lifestyle factors (smoking, alcohol and physical activity). These factors were considered as covariates as they have been associated with work disability (e.g.,Salonsalmi et al. 2012; Lallukka et al. 2014, 2015; Ervasti et al. 2017; Svärd et al. 2018). For descriptive purposes, *age* was categorized as follows: 19‒29 years, 30‒34 years and 35‒39 years. *Gender* was a dichotomous variable (woman vs man). The main analysis used a list of self-reported *medically confirmed chronic conditions* that are likely to affect work ability (asthma, sleep apnea, osteoarthritis, hypertension, other heart or vascular disease, diabetes, eating disorder, depression, anxiety disorder, other mental disorder and migraine). For descriptive purposes, the following categories were used: 0, 1 or ≥ 2 diseases. Data on acute/subacute and chronic *pain* were collected. The employees were first asked whether they were currently experiencing any pain and then separately about the duration of pain. Following the conventional classifications (Merskey 1986), pain lasting up to 3 months was defined as acute/subacute pain, and pain lasting more than 3 months was defined as chronic pain. *Body mass index (BMI)* was calculated as weight in kilograms divided by height in meters squared, using self-reports and dichotimized into non-obese (≤ 30 kg/m^2^) and obese (> 30 kg/m^2^). *Sleep problems* were measured by a 4-item version of the Jenkins questionnaire (Jenkins et al. 1988) and those participants reporting at least one of the four symptoms (trouble falling asleep, wake up several times per night, trouble staying asleep, feeling tired after usual amount of sleep) occurring more than 14 days a month were classified as having sleep problems (Lallukka et al. 2020a). Based on the regular *smoking habits* the participants were categorized as non-, current, or former smokers. *Binge drinking* was measured by the frequency of having consumed more than six units of alcohol on one occasion, and it was dichotomized as no binge drinking (less than once a month for women and less than once a week for men) and binge drinking (more than once a month for women and once a week for men). Weekly hours of *physical activity* during commuting and leisure time and their intensity levels within the previous 12 months were also assessed. Approximate metabolic equivalent task (MET) hours per week were calculated by multiplying the time spent in physical activity with the MET value of each intensity level and adding these up. For the purposes of this study, we categorized physical activity into inactive (≤ 14 MET hours/week) and active (> 14 MET hours/week) (Lahti et al. 2010).

### Statistical analysis

Characteristics of the participants are reported as percentages and mean values of sickness absence days, by baseline characteristics, with 95% confidence intervals (CI). Other descriptive statistics are presented as means and their standard deviations (SD). Negative binomial regression analysis was applied to examine the rate of sickness absence during the follow-up in relation to the physical work factors. Natural logarithm of the follow-up time was included in the models as an offset variable to consider the follow-up times of unequal length. All models were adjusted for age and gender. Smoking, binge drinking and physical activity (model 2), and chronic conditions, pain, obesity and sleep problems (model 3) were adjusted for in further models. In models 1–3, the associations were analyzed separately for each working condition, and in model 4 all working conditions were mutually adjusted for. Results are presented as rate ratios (RRs) with their 95% CIs. In addition, to demonstrate the absolute level of work disability of the different work exposure groups, the least square means per 10 person-years adjusted for age and gender were calculated. Potential interactions between each occupational exposure and gender on sickness absence were tested. SAS statistical software, version 9.4 (SAS Institute, Cary, North Carolina), was used for all analyses.

## Results

The mean age of the participants was 32.0 years (SD 4.6), and about two thirds of the participants were under 35 years of age. Half of the participants had one or more chronic conditions, and 43% reported experiencing subacute, acute, or chronic pain. Mean BMI was 25.3 (SD 5.2), and 15% of the participants were categorized as obese (BMI ≥ 30). Approximately one third of the participants reported suffering from sleep problems, one fourth were current smokers, and a fifth of the participants engaged in binge drinking (Table 1.).

**Table 1:** Baseline characteristics of participants (*n* = 3542) and mean number of sickness absence days (mean, 95% Confidence Interval, CI) during 12-month follow-up

Baseline characteristic	Distribution (%)*			Number of sickness absence days/10 person-years*	
	All *n* = 3542	Women *n* = 2784	Men *n* = 758	Mean	95% CI
All	100			212	202–224
Women	79			231	217–245
Men	21			146	130–164
Age					
19–29	32	34	26	251	229–276
30–34	33	33	33	203	185–223
35–39	35	33	41	187	171–205
Number of chronic conditions					
0	49	46	58	153	142–165
1	29	31	26	225	204–247
** ≥ **2	22	23	16	332	296–371
Pain					
No	57	55	65	162	151–173
Subacute/acute	23	24	18	262	235- 292
Chronic	20	21	17	304	270–342
Obesity					
No	85	85	85	195	184–207
Yes	15	15	15	313	273–359
Sleep problems					
No	70	69	76	175	164–186
Yes	30	31	24	303	275–334
Smoking					
Never-smoker	54	56	49	187	173–200
Ex-smoker	21	20	25	216	192–243
Current smoker	25	24	26	267	240–297
Binge drinking					
No	81	80	83	207	195–220
Yes	19	20	17	234	208–265
Physically inactive (MET ≤ 14)					
No	89	90	88	208	196–220
Yes	11	10	12	253	215–298

*Unadjusted

The mean follow-up time of sickness absence days was 339 days (SD 74.9), and 86% of the participants were followed up for 365 days. Fourteen percent (*n* = 484) of the participants had a follow-up time less than 365 days due to the end of the employment contract and the mean follow-up time among them was 177 days (SD 103.1). During the follow-up, participants had on average 212 sickness absence days per 10 person-years (Table 1). The number of sickness absence days was higher among women than among men, and it was the highest among the youngest age group. In addition, participants with chronic conditions and those with pain, obesity or sleep problems at baseline had more sickness absence days during the follow-up. Poor health-related lifestyle was also associated with a higher number of subsequent sickness absence days (Table 1).

**Fig. 1.**
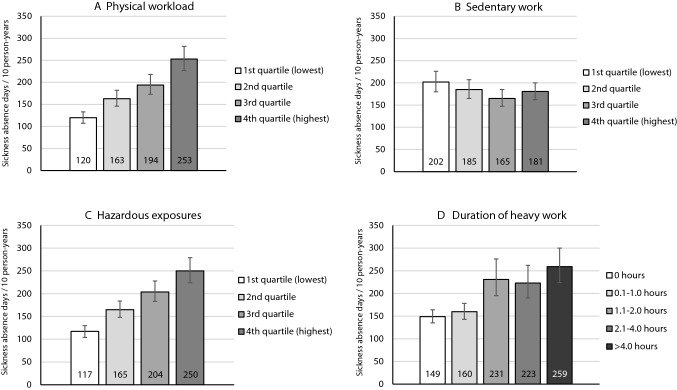
Number of sickness absence days/10 person-years by work exposures: **A** physical workload, **B** sedentary work, **C** Hazardous exposures and **D** duration of heavy physical work per day. Adjusted for age and gender

Figure 1 shows the age and gender adjusted number of sickness absence days by different work exposures. Higher exposure to physical workload or hazardous exposures, and longer daily exposure to heavy physical work were associated with a higher number of sickness absence days during the follow-up, whereas higher exposure to sedentary work tended to be associated with a lower number of sickness absence days (Fig. 1). RRs are presented in Table 2, Model 1. For example, RRs for sickness absence among participants whose exposure to physical workload or hazardous exposures was in the highest exposure quartile were 2.1 (95% CI 1.8‒2.5) and 2.2 (95% CI 1.9‒2.5), respectively, compared with those whose exposure was in the lowest quartile. In addition, RRs for sickness absence among participants who reported that they do heavy physical work 1.1‒2.0 h, 2.1‒4.0 h or over 4 h daily were 1.6 (95% CI 1.3‒1.9), 1.5 (95% CI 1.3‒1.8) and 1.7 (95% CI 1.5‒2.1), respectively, compared with those who reported no physical work. For sedentary work, the observed associations tended to be to the opposite direction and participants in the second highest exposure tertile had the lowest risk for sickness absence (RR 0.82, 95% CI 0.70–0.95). However, these associations were not strong. After adjusting further for lifestyle factors and health characteristics, these associations attenuated only slightly (Table 2, Models 2‒3). The mutual adjustment for all physical working conditions attenuated the observed associations somewhat but they remained statistically significant for physical workload and hazardous exposures (Table 2).

**Table 2:** Rate ratios (RRs, 95% confidence intervals) for sickness absence days during the 12-month follow-up by work exposures

Work exposure	%	Model 1*		Model 2*		Model 3*		Model 4*	
		RR	95% CI	RR	95% CI	RR	95% CI	RR	95% CI
Physical workload									
1st quartile (lowest)	25	1		1		1		1	
2nd quartile	25	1.36	1.17–1.57	1.36	1.18–1.58	1.30	1.12–1.50	1.21	1.04–1.42
3rd quartile	25	1.62	1.39–1.88	1.60	1.39–1.87	1.49	1.29–1.72	1.32	1.12–1.57
4th quartile (highest)	25	2.11	1.82–2.45	2.05	1.76–2.38	1.75	1.51–2.03	1.52	1.24–1.85
Sedentary work									
1st quartile (lowest)	25	1		1		1		1	
2nd quartile	25	0.92	0.78–1.06	0.90	0.78–1.05	0.94	0.82–1.09	1.04	0.89–1.21
3rd quartile	25	0.82	0.70–0.95	0.82	0.70–0.95	0.83	0.72–0.96	0.98	0.84–1.15
4th quartile (highest)	25	0.90	0.77–1.04	0.90	0.78–1.06	0.89	0.77–1.03	1.09	0.93–1.29
Hazardous exposures									
1st quartile (lowest)	25	1		1		1		1	
2nd quartile	25	1.42	1.23–1.65	1.41	1.21–1.63	1.39	1.20–1.60	1.31	1.13–1.53
3rd quartile	25	1.75	1.51–2.03	1.75	1.51–2.02	1.56	1.35–1.80	1.55	1.33–1.81
4th quartile (highest)	25	2.15	1.85–2.49	2.14	1.84–2.48	1.85	1.60–2.14	1.70	1.45–2.01
Heavy physical work (hours/day)									
0.0 h	39	1		1		1			
0.1–1.0 h	26	1.07	0.94–1.23	1.07	0.94–1.22	1.06	0.93–1.20	0.95	0.82–1.09
1.1–2.0 h	9	1.55	1.28–1.88	1.57	1.29–1.89	1.42	1.18–1.71	1.18	0.96–1.46
2.1–4.0 h	12	1.50	1.25–1.78	1.44	1.21–1.72	1.34	1.13–1.59	1.12	0.92–1.38
> 4 h	14	1.74	1.47–2.05	1.65	1.40–1.96	1.53	1.30–1.80	1.22	1.00–1.51

Model 1 adjusted for age and gender

Model 2 adjusted for Model 1 + smoking, binge drinking and leisure-time physical activity

Model 3 adjusted for Model 1 + the number of chronic conditions, pain, obesity and sleep problems

Model 4 adjusted for age and gender + all four work exposures

*In Models 1–3 the associations were analyzed separately for each working condition. In Model 4 all working conditions were mutually adjusted for

A statistically significant interaction between gender and one of the occupational exposures (physical workload) on sickness absence was observed, but due to the small number of men in the analytical sample (*n* = 758, 21%) (Table 1) women and men were pooled in the main analyses. However, gender stratified results between physical workload and sickness absence are presented in Supplement 1 and Supplement 2. No significant gender interactions were observed for the other occupational exposures.

## Discussion

The present study showed that adverse physical occupational exposures were associated with sickness absence among 19–39-year-old municipal employees in a dose–response manner: higher exposure, more sickness absence days. In contrast, a higher amount of sedentary work tended to be associated with less sickness absence days.

Our findings on the association between physically demanding work exposures and subsequent sickness absence are in line with previous studies (Christensen et al. 2008; Laaksonen et al. 2010b; Saastamoinen et al. 2014; Gupta et al. 2020; Halonen et al. 2021); however, they extend prior research by providing new evidence of the associations among younger, under 40-year-old employees. The present results have important implications for the prevention of work disability. By understanding the factors affecting health and wellbeing already at the early and mid-career, the results can help detect the high-risk groups for work disability early enough to reduce sickness absence and postpone employees’ permanent withdrawal from the labor market. Many of the previous studies have had a narrower focus examining the effect of physical workload only, overlooking the possible effects of other physical working conditions. Moreover, previous studies have not been able to evaluate the effect of duration of daily occupational exposure on work disability. In the current study, we were able to consider the effects of various environmental hazards and sedentary work on sickness absence and demonstrate how the duration of daily exposure can influence the associations between physically heavy work and sickness absence.

In addition to heavy physical work, occupational environment often limits employees’ physical activity and it has been suggested that sedentary work may also be associated with adverse health outcomes (Sjöström et al. 2006; Owen et al. 2010; van Uffelen et al. 2010; Shrestha et al. 2015; Mänty et al. 2016). However, previous studies exploring the association between sedentary work and sickness absence are scarce (Laaksonen et al. 2010b; Lallukka et al. 2019). In the present study, a higher amount of sedentary work tended to be associated with less sickness absence. However, the observed associations were modest and statistically significant only for the participants in the second highest exposure quartile. Furthermore, as our study was observational and relied on self-reported exposure data, conclusions about possible protective effects cannot be drawn. The effects of sedentary work need to be explored more in detail in future studies.

There is strong evidence indicating socioeconomic differences in sickness absence: the lower the socioeconomic position, the higher the risk for sickness absence (Melchior et al. 2005; Piha et al. 2007; Christensen et al. 2008; Hansen and Ingebrigtsen 2008; Löve et al. 2013; Pekkala et al. 2017). Similar socioeconomic differences have been observed in studies focusing on young employees (Sumanen et al. 2015a, 2016, 2017a). When considering the results of the current study, it is important to acknowledge these socioeconomic differences in sickness absence. However, as socioeconomic status is strongly associated with the presence or absence of adverse physical working conditions (Laaksonen et al. 2010a), we did not adjust our analysis for socioeconomic position to avoid overadjustment.

The strengths of the study include longitudinal design with reliable register-based outcome data. In addition, we used a well-characterized occupational cohort which included participants from different occupational classes and a sufficient variety of work exposures. Our measure of physical working conditions allowed us to consider how different physical occupational exposures are associated with subsequent work disability.

One limitation of the study is that we studied a rather small sample of public sector occupational cohort with mostly female participants. This limits the generalizability of our results. The size of the analytical sample was affected by the response rate, not giving consent to record linkages, and missing data on either the exposures or the covariates. However, the included participants and those who gave their permission to register linkages had the same amount of sickness absence days during the 12-month post-survey follow-up. In addition, working conditions were measured only at baseline and the study could have benefited from repeated measurements during the follow-up. This would have enabled assessment of possible changes in the exposures. However, as the follow-up was relatively short, it is likely that the effects of any changes in working conditions would have been relatively small. Furthermore, even though we adjusted for several sociodemographic and health characteristics as well as lifestyle factors, the results may have been affected by unmeasured and residual confounding. Finally, our study focused on all-cause sickness absence, and future studies need to address the diagnosis specific work disability outcomes. In particular, associations between physical working conditions and sickness absence due to mental disorders should be assessed as mental disorders are the main cause of work disability in young employees.

## Conclusions

Exposure to physically demanding work was associated with a higher number of sickness absence days among 19–39-year-old municipal employees. Sedentary work was associated with less sickness absence. Physical working conditions should be considered when aiming to support work ability.

## Supplementary Information

Below is the link to the electronic supplementary material.

Supplementary file1 (DOCX 21 kb)
