# Supplementary material for: Physical working conditions and subsequent sickness absence: a record linkage follow-up study among 19–39-year-old municipal employees
Source: Int Arch Occup Environ Health. 2021 Oct 23;95(2):489–97. doi: 10.1007/s00420-021-01791-y (PMC8795030; doi:10.1007/s00420-021-01791-y)
Supplement: Supplementary file 1 — Supplementary file1 (DOCX 21 kb) [file 420_2021_1791_MOESM1_ESM.docx]

**Supplement 1.** Number of sickness absence days / 10 person-years by physical workload by gender. Adjusted for age.

**Supplement 2.** Rate ratios (RRs, 95% confidence intervals) for sickness absence days during the 12-month follow-up in relation to physical workload by gender.

| **Work exposure** | **Model 1** | |  | **Model 2** | |  | **Model 3** | |  |
| --- | --- | --- | --- | --- | --- | --- | --- | --- | --- |
|  | **RR** | **95% CI** |  | **RR** | **95% CI** |  | **RR** | **95% CI** |  |
| **Physical workload, *Women*** |  |  |  |  |  |  |  |  |  |
| 1^st^ quartile (lowest) | 1 |  |  | 1 |  |  | 1 |  |  |
| 2^nd^ quartile | 1.31 | 1.11-1.54 |  | 1.33 | 1.32-1.57 |  | 1.29 | 1.10-1.51 |  |
| 3^rd^ quartile | 1.42 | 1.21-1.68 |  | 1.43 | 1.21-1.68 |  | 1.37 | 1.17-1.61 |  |
| 4^th^ quartile (highest) | 2.05 | 1.74-2.42 |  | 2.01 | 1.70-2.36 |  | 1.70 | 1.44-2.00 |  |
| **Physical workload, *Men*** |  |  |  |  |  |  |  |  |  |
| 1^st^ quartile (lowest) | 1 |  |  | 1 |  |  | 1 |  |  |
| 2^nd^ quartile | 1.60 | 1.11-2.30 |  | 1.47 | 1.02-2.13 |  | 1.35 | 0.94-1.93 |  |
| 3^rd^ quartile | 2.54 | 1.76-3.67 |  | 2.45 | 1.70-3.54 |  | 2.03 | 1.41-2.92 |  |
| 4^th^ quartile (highest) | 2.41 | 1.67-3.47 |  | 2.25 | 1.56-3.25 |  | 1.97 | 1.38-2.85 |  |

**Model 1** adjusted for age

**Model 2** adjusted for Model 1 + smoking, binge drinking and leisure-time physical activity

**Model 3** adjusted for Model 1 + the number of chronic conditions, pain, obesity and sleep problems
